# Comparative Analysis of Hemagglutination Inhibition Titers Generated Using Temporally Matched Serum and Plasma Samples

**DOI:** 10.1371/journal.pone.0048229

**Published:** 2012-12-20

**Authors:** Gabriel N. Defang, Nicholas J. Martin, Timothy H. Burgess, Eugene V. Millar, LeNae A. Pecenka, Janine R. Danko, John C. Arnold, Tadeusz J. Kochel, Thomas C. Luke

**Affiliations:** 1 Viral and Rickettsial Diseases Department, Naval Medical Research Center, Silver Spring, Maryland, United States of America; 2 Department of Preventive Medicine and Biometrics, Infectious Disease Clinical Research Program, Uniformed Services University of the Health Sciences, Bethesda, Maryland, United States of America; 3 Pediatrics and Infectious Diseases, Naval Medical Center, San Diego, California, United States of America; Duke-NUS Gradute Medical School, Singapore

## Abstract

Influenza-specific hemaggluitination inhibition (HAI) antibody titer, an indicator of immunity to influenza, is often used to measure exposure to influenza in surveillance and immunogenicity studies. Traditionally, serum has been the specimen of choice for HAI assays, but a desire to reduce the amount of blood collected during studies and the availability of plasma in archived sample collections warrant the evaluation of plasma for HAI titer. Therefore, the relationship between serum and plasma HAI titer values is of great interest. Here, we compare HAI titers determined on temporally matched serum and plasma (citrated and heparinized) using influenza A and B viruses. Bland-Altman plots, McNemar's test, and geometric coefficient of variation were used respectively for evaluating agreement, correlation and variability in the serum-plasma titer results. We observed a high degree of agreement (80.5%–98.8%) and correlation (r = 0.796–0.964) in the serum and matched plasma titer values although plasma titers were generally lower than corresponding serum titers. Calculated seropositive (HAI ≥40) rates were higher using serum titers than with plasma titers, but seroconversion rates were unaffected by sample type. Stronger agreement and decreased variability in titers were seen between serum and citrated plasma than between serum and heparinized plasma. Overall, these data suggest that serum or plasma can be used in serodiagnostic HAI assays, but seropositive rates may be underestimated using plasma HAI titers. The type of anticoagulant present in plasma may affect HAI titer values and warrants further investigation.

## Introduction

The influenza hemagglutination-inhibition (HAI) assay first described in the 1940's (Hirst 1942, Salk 1944) is the traditional method for measuring immune responses to influenza virus hemagglutinin (HA), the principal antigen relevant to protection. The HAI assay is used extensively for evaluation of influenza vaccine efficacy and in epidemiological studies of influenza virus infection. Mechanistically, the assay capitalizes on the fact that HA glycoproteins on the surface of influenza virions bind and agglutinate erythrocytes. The attachment of serum antibodies to specific epitopes on the HA glycoprotein interferes with virus binding to receptors on the erythrocytes, inhibiting agglutination. Historically, serum has been used in the performance of HAI [1], [2], [3], [4]. However, increasingly in human subject research studies, plasma is a preferred and more frequently collected specimen type compared to serum [5], [6], [7]. This is due in part to the near universality of plasma as the specimen of choice for measuring several analytes in human samples coupled with blood volume constraints imposed on human subject research. It is therefore of interest to compare HAI activity levels in serum and plasma. This is especially true in retrospective epidemiological studies seeking to chronicle a newly emergent influenza strain in an affected region when previously collected plasma is the only sample type available for testing. In such cases the validity of plasma HAI antibody titers will come into question.

We have recently collected high-titered plasma units from influenza convalescent individuals and vaccine recipients for use in a randomized, multicenter study to explore the efficacy of convalescent plasma therapy as an alternate treatment modality for severe influenza disease. In order to determine and/or confirm the HAI titers of plasma units to be used in immunotherapy, serum from unit donors were tested. However, it may be more practical to directly test the plasma units on the hospital blood bank shelves to determine acceptability prior to infusion into patients.

Anticoagulants present in plasma are known to interfere with antibody-antigen reactions and may inhibit the activity of some enzyme reagents [8], [9]. For these reasons plasma has traditionally not been considered the specimen of choice for assays that either measure antibodies or require enzyme reagents. In the case of influenza HAI, anticoagulants may interfere with binding of antibodies to the HA molecule of the virus, or hinder enzyme activity during the elimination of non-specific inhibitors of agglutination in test samples. Nonetheless, there are published reports of HAI titers obtained from plasma samples [6], [7]. Other investigators have reported HAI titer values derived from a combination of serum and plasma samples [5], though unfortunately a detailed comparison of the serum and plasma HAI titers was not presented. To date, no detailed account elucidating the HAI titer difference between temporally matched serum and plasma has been reported. Additionally, the impact of anticoagulant selection on plasma HAI results has not been examined.

The purpose of this study was to evaluate the correlation and agreement of HAI antibody titers of temporally matched serum and plasma samples and to ascertain if plasma can be used in place of serum in standard influenza HAI testing. We also assessed the effect of anticoagulants in HAI assay variability. Five influenza virus strains and two distinct anticoagulated plasma were used in this study to evaluate potential differences in HAI titer values associated with these variables.

## Materials and Methods

### Ethics Statement

The Infectious Diseases Institutional Review Board (IDCRP# -046 and IDCRP# -045) and Naval Medical Research Center Institutional Review Board (NMRC.2010.0012 and NMRC.2010.0004) approved the informed consent and sample collection protocols for this study. All participants provided written informed consent to participate in the study. Sample collections under the two study protocols were in compliance with all applicable Federal regulations governing the protection of human subjects.

### Samples

Serum and plasma samples were collected during two separate studies. One-hundred and sixty-five serum and plasma (sodium citrate anticoagulant) samples were collected simultaneously from volunteers during organized blood drives at Department of Defense (DoD) installations during the period of November 2009 through June 2011. One-hundred and forty-nine paired serum and plasma (lithium heparin anticoagulant) samples were collected on visit 1 (day 0) and visit 4 (day 28) from individuals presenting with influenza-like illness (ILI) symptoms with duration of ≤48 hours under the Acute Respiratory Infection Consortium longitudinal study of the natural history of ILI in DoD beneficiaries.

### Hemagglutination Inhibition Assay

Influenza antibody titers were detected using HAI with 2-fold serial dilutions, as described elsewhere [2], [10], [11], at the Naval Medical Research Center (Silver Spring, MD). HAI assays were conducted using 0.5% turkey erythrocytes; the reference antisera were supplied by the Centers for Disease Control and Prevention. A/California/07/2009 NYMC X-179A (H1N1pdm), A/Brisbane/59/2007 IVR-148 (sH1N1), A/Victoria/210/2009 NYMCX-187 (sH3N2-2009), A/Uruguay/716/2007 X-175C (sH3N2-2007) and B/Brisbane/60/2008 (Inf B) viruses were used in the assays. Serum and plasma samples were treated at a 1∶3 ratio (vol/vol) with receptor destroying enzyme (Denka Seiken Co., Tokyo, Japan) and incubated at 37°C for 18–20 hr. The RDE was inactivated at 56°C for 45 min, followed by addition of 6 volumes of PBS (pH 7.2) resulting in an initial testing dilution of 1∶10. RDE-treated serum and plasma samples were further heme-adsorbed at a 4∶1 ratio (vol/vol) with packed turkey derived RBCs (Lampire Biological Products, Pipersville, PA). HAI assays were performed in Nunc V-bottom 96-well microtiter plates (Nalge Nunc International, Rochester, NY). Standardized virus (25 µL; 16 HA units/100 µL) was added to 25 µL of serially diluted (two fold) test sera and plasma, mixed, and incubated at room temperature for 30 min. Following incubation, 50 µL of standardized turkey RBCs (Lampire Biological Products, Pipersville, PA) was added to all wells. Plates were observed for agglutination after 30 min by tilting at a 45° to 60° angle. The reciprocal of the highest dilution of serum or plasma that completely inhibited hemagglutination was determined to be the HAI titer. Samples were tested in duplicate in two independent assays, with geometric mean titer (GMT) reported as the final titer. For computational purposes, titers of <1∶10 were assigned a value of 1∶5, and those >1∶1280 were assigned a value of 1∶1280.

### Statistical Analysis

The geometric mean, standard deviation, and coefficient of variation were calculated using the method described by Kirkwood [12]. Titer values were log_2_ transformed prior to conducting paired t-test analysis of serum and plasma results for each virus. McNemar's test was used to evaluate the correlation between the number of seropostive (HAI >40) samples detected in serum and plasma samples. This approach was repeated for the evaluation of high-titer (HAI >160), increased HAI titer values from Visit 1 (acute) to Visit 4 (convalescent), and seroconversion (4-fold increase in acute to convalescent HAI titer) in serum and plasma samples. The Pearson Product-moment Correlation Coefficient was calculated to assess the correlation between matched serum and plasma HAI titer values. A *p* value of less than 0.05 was considered to indicate statistical significance.

The agreement between matched serum and plasma samples was assessed using a method described by Bland and Altman [13]. The mean GMT was plotted on the X-axis and the dilution factor difference between the serum and plasma values was plotted on the Y-axis. Y values >1 indicate a difference in the dilution factor larger than one.

## Results

### Comparative HAI titers using serum and sodium citrated plasma

The HAI assay was conducted on 165 temporally matched serum and sodium citrated plasma samples using 2 H1N1 strains, 2 H3N2 strains and 1 influenza B strain (Table 1). All serum and plasma samples were tested in duplicate in the same assay. The HAI GMT values observed using plasma were generally lower than the corresponding serum values, with statistically significant differences (*p*<0.05; paired t-test of log_2_ transformed results) observed between serum and plasma HAI GMT values for four of the five viruses tested (Table 1). The serum and plasma data were further analyzed to determine the number of seropositive (HAI titer >40) samples, as well as the number of high-titer (HAI titer >160) samples; a key parameter for identifying plasma units for experimental influenza immunotherapy. There was significant discordance (*p*<0.05, McNemar test) in the identification of seropositive samples between serum and plasma when H1N1pdm and sH1N1-2007 viruses were used. There was also a significant discordance in the identification of high-titer samples between serum and plasma with H1N1pdm and sH3N2-2007 viruses.

**Table 1:** Comparison of HAI data for 165 paired serum-citrated plasma samples for five influenza viruses.

	Sample Type	GMT (GSD)	Percent of Samples with HAI >40 (n)	Percent of Samples with HAI >160 (n)
**H1N1pdm**	serum	52.12 (4.58)†	65.5 (108)	30.3 (50)
	plasma	41.63 (4.47)	60.0 (99) ‡	27.3 (45) ‡
**sH3N2-2009**	serum	22.59 (3.74)†	37.6 (62)	10.3 (17)
	plasma	20.90 (3.50)*	36.4 (60)	9.7 (16)
**sH3N2-2007**	serum	23.27 (1.56)†	40.6 (67)	15.2 (25)
	plasma	20.60 (3.96)	37.6 (62)	9.7 (16) ‡
**sH1N1**	serum	20.77 (3.55)	36.4 (60)	10.3 (17)
	plasma	19.92 (4.23)	32.7 (54) ‡	8.5 (14)
**Inf B**	serum	7.89 (2.22)†	7.3 (12)	1.2 (2)
	plasma	7.27 (2.09)	6.7 (11)	0.6 (1)

† p<0.05 for T-test of log_2_ transformed data.

‡ p<0.05 for McNemar Test.

**Figure 1:**
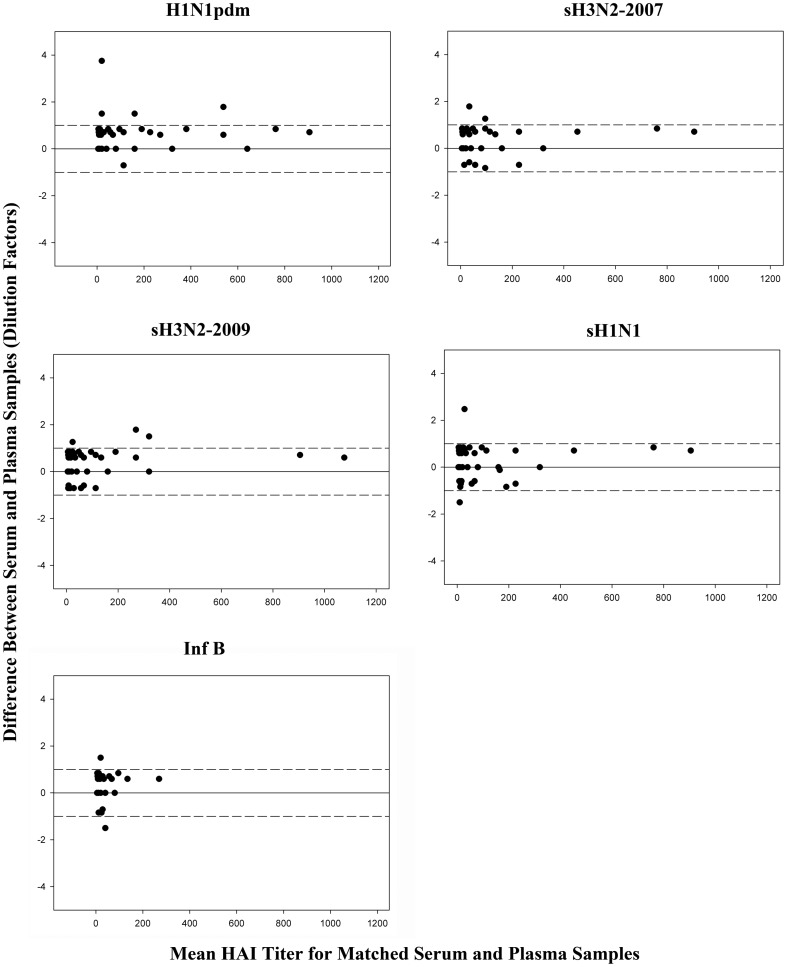
Bland-Altman Plots for 165 serum-citrated plasma samples. Dashed lines denote the limit of agreement (all HAI values within 1 dilution factor). Overall agreement between the serum and plasma samples was ≥97%.

Since intra-laboratory variations in standard HAI titer values within one dilution factor are a common and acceptable result of multiple testing, serum and matched plasma HAI titers were evaluated to determine the degree of agreement (within one dilution factor) using Bland-Altman plots. The plots (Figure 1) indicated a strong agreement irrespective of the virus used in the assay. For the corresponding test viruses, there was 97.6% (H1N1pdm), 98.2% (sH3N2-2009) and 98.8% (sH3N2-2007, sH1N1-2007, and Inf B) agreement between serum and plasma HAI titers.

### Comparative HAI titers using paired serum and lithium heparinized plasma

The HAI assay was conducted on 149 temporally matched serum and lithium heparinized plasma using 1 H1N1 strain, 1 H3N2 strain and 1 influenza B strain (Table 2). All paired (visit 1 and 4) serum and plasma samples were tested in duplicate in the same assay. Consistent with the citrated plasma samples the HAI titer values using heparinized plasma were generally lower, and underestimated the titer values obtained using matched serum samples. Visit 1 (v1) and 4 (v4) serum group GMTs were higher than matched plasma group GMTs regardless of the virus strain used in the assay (Table 2). The paired serum and plasma samples were further analyzed to determine the comparative number of seropositive (HAI titer >40) samples, as well as the comparative number of seroconversions (4 fold rise in titer between v1 and v4). There was significant discordance (*p<0.05*, McNemar test) in the identification of seropositive samples between v1 sample types when H1N1pdm was used, and between v1 and v4 sample types when sH3N2-2009 virus was used. On the other hand, regardless of the virus used in the HAI assay, no significant difference was observed in the seroconversion rates when the matched plasma and serum data sets were compared (Table 2).

**Table 2:** Comparison of HAI data from Visit 1 (acute phase of ILI-symptoms) and Visit 4 (convalescent phase of ILI-symptoms) for 149 paired serum-heparinized plasma samples for three influenza viruses.

	Sample Type	Visit 1 GMT (GSD)	Percent of Visit 1 Samples with HAI >40 (n)	Visit 4 GMT (GSD)	Percent of Visit 4 Samples with HAI >40 (n)	Percent of Visit 4 Samples with 4-fold Increase in HAI Values Compared to Visit 1 (n)
**H1N1pdm**	serum	59.38 (6.31)†	59.1 (88)&	67.78 (1.61)†	64.4 (96)‡	6.7 (10)
	plasma	41.69 (5.65)†	54.0 (80)‡	51.40 (5.40)	59.7 (89)‡	9.4 (14)
**sH3N2-2009**	serum	38.51 (4.35)†	54.0 (80)‡	48.79 (4.40)†	59.7 (89)‡	8.7 (13)
	plasma	25.43 (4.02)$	42.3 (63)‡	34.18 (4.52)	50.3 (75)‡	9.4 (14)
**Inf B**	serum	15.16 (2.64)	20.8 (31)‡	14.74 (2.56)	10.7 (16)‡	3.4 (5)
	plasma	14.34 (2.72)	20.1 (30)‡	15.51 (2.78)	10.7 (16)‡	3.4 (5)

† p<0.05 for T-test of log_2_ transformed data.

‡ p<0.05 for McNemar Test.

**Figure 2:**
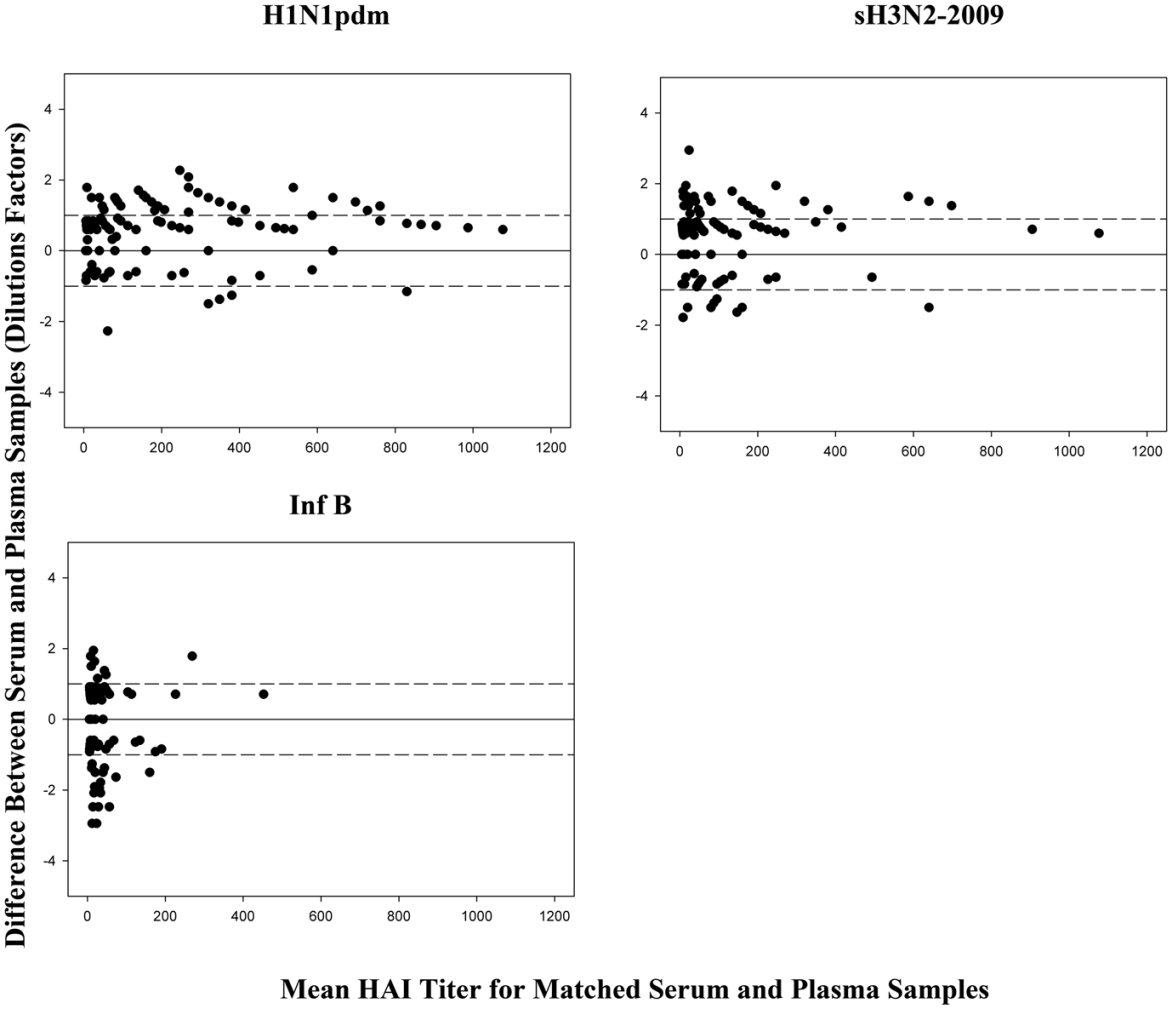
Bland-Altman Plots for 149 paired serum-heparinized plasma v1 and v4 samples. Dashed lines denote the limit of agreement (all HAI values within 1 dilution factor). Overall agreement between the serum and plasma samples was ≥80%.

Paired serum and matched plasma HAI titers were evaluated to determine the degree of agreement (within one dilution factor) using Bland-Altman plots. The plots (Figure 2) indicated that there was 84.6% (H1N1pdm), 83.2% (sH3N2-2009) and 86.6% (Inf B) agreement between serum and plasma HAI titers.

### HAI variability with heparinized or citrated plasma

We evaluated the impact of anticoagulant type on the variability of plasma titers compared to matched standard serum titers. The HAI titer variability in the 165 paired serum and matched citrated plasma data set was compared to that of the 149 paired serum and matched heparinized plasma. This comparison was conducted by evaluating the geometric coefficient of variance (GCV) in the titer values for each of the two serum–anticoagulated plasma sample sets, as well as the correlation and agreement between serum and plasma samples.

The GCV observed in serum and plasma samples was generally lower in the serum-citrated plasma samples than the heparinized plasma samples (data not shown). This indicates the HAI values were more varied in the heparinized plasma samples than in the citrated plasma samples. The correlation of plasma HAI values to serum HAI values was assessed by calculating the Pearson Product-Moment Correlation Coefficient for the serum-plasma sample sets (Table 3). Serum and plasma HAI values were highly correlated (>0.80) for all viruses tested. The degree of correlation between serum and plasma samples was greater with serum-citrated plasma HAI titer values for sH3N2-2009 and Inf B. A higher proportion of serum-citrated plasma samples tested had HAI titer values within one dilution factor indicating a greater degree of agreement than that observed in the serum-heparinized plasma samples.

**Table 3:** Correlation and Agreement for two serum-anticoagulated plasma sample sets.

		Serum-citrated plasma	Serum-heparinized plasma
**H1N1pdm**	Correlation (r)	0.876	0.891
	Agreement	97.6%	80.5%
**sH3N2-2009**	Correlation (r)	0.964	0.943
	Agreement	98.2%	82.6%
**Inf B**	Correlation (r)	0.951	0.796
	Agreement	98.8%	88.3%

## Discussion

The HAI assay serves as an important tool in the characterization of influenza immune responses. The serum HAI antibody titer is often used as a surrogate marker of protection and is an important immunogenicity measure in humans [14], [15], [16]. Increasingly, plasma has been used as a convenient sample in HAI assays, but the degree of correlation and agreement to standard serum HAI titers has not been fully described. Here, we observed a high degree of agreement in HAI titers when serum and temporally matched plasma samples were used in HAI assays.

Generally, intra-laboratory variability in HAI titer values within one dilution factor from duplicate or repeated testing of a given sample is considered acceptable and the values are comparable. Therefore, in this study, agreement was defined as serum and matching plasma titer values within one dilution of each other. Using this criterion, agreement between serum and matched plasma samples ranged from 80.5% to 98.8% (Figures 1 and 2). The degree of agreement appeared to be related to the type of anticoagulant present in the matched plasma samples with higher correlation (>97%) observed in serum and matched citrated plasma samples than the serum and matched heparinized plasma (80–88%) for all viruses tested. Based on this observation, we believe the citrated plasma could be used in place of serum samples to measure HAI titer values.

Despite the high degree of agreement between matched samples, there were statistically significant differences observed at the group level. Generally, the plasma GMT values were lower than their matched serum GMT values (Tables 1 and 2), but were still within acceptable range irrespective of virus or anticoagulant used in the assay (Tables 1 and 2). There were a few instances were individual plasma titers were higher than matching serum titers, nonetheless, the likelihood of getting higher serum titer values compared to matching plasma titers was greater in the sample set used in this study. As a consequence, a greater number of samples were identified as seropositive or high-titered by serum HAI testing than by plasma HAI testing for each of the four influenza A viruses used (Tables 1 and 2). Comparable seropositive numbers were seen with influenza B. On the other hand, sample type did not affect seroconversion rates as HAI testing of paired sera and matching plasma identified similar numbers of seroconverts (Table 2).

The observation that plasma titers were lower than matching serum titers has implications to the use of plasma for HAI analysis in seroprevalence and seroconversion studies. A primary concern is the potential for underestimating influenza seroprevalence rates when plasma samples are used in HAI antibody testing. This concern needs to be balanced with the need to characterize the emergence of a novel influenza strain in previously collected plasma samples. The potential to underestimate the seroprevelance may be an acceptable limitation in the design of retrospective epidemiological studies with the primary aim of determining the temporal and population dynamics following the emergence of a novel influenza virus. Another concern related to the tendency of plasma HAI titers to underestimate serum HAI titers is the impact of sample type on HAI values used to measure vaccine effectiveness. New vaccine candidates are expected to meet specific seropositive and seroconversion rate thresholds, such as those outlined in the FDA guidelines on clinical data needed to demonstrate effectiveness and support pandemic vaccine licensure [17], here, the use of plasma samples may put effectiveness studies at a disadvantage to meet set thresholds.

The reasons for the lower plasma titers are unknown and were not investigated further in this study. However, we hypothesize that anticoagulant activity affects HAI values and is responsible for the overall lower comparative plasma titers. Heparin present in some of the plasma samples used in this study is known to decrease antibody reaction rate and interferes with antibody-antigen reaction [8], [9]. Sodium citrate present in the paired plasma samples is a chelating agent and can bind enzyme cofactors which may affect enzyme activity in assays. Additionally, occasional plasma clots that occur in some plasma samples can affect assay readout with a bias towards lower titer readouts as clots could mimic agglutination patterns in affected wells. The observed difference in the degree of agreement when sodium citrated plasma values versus heparinized plasma values were compared with matching serum values was interesting (Table 3). It is possible that the variation may be related to anticoagulant type or the varying concentrations of anticoagulant from sample to sample, however, further investigation is needed to rule out chance occurrence.

The high degree of agreement in HAI values observed between individual serum and matched plasma samples suggests that plasma could be used in serodiagnostic HAI assays. Serum or plasma can be used for determining flu-specific antibody titers in epidemiological studies, with the caveat that seropositive rates could be underestimated with use of plasma in these population based studies. At this time the effects of different anticoagulants in HAI assays is not well characterized and warrants further investigation.
